# Predictors of good response to conventional synthetic DMARDs in early seronegative rheumatoid arthritis: data from the ESPOIR cohort

**DOI:** 10.1186/s13075-019-2020-x

**Published:** 2019-11-15

**Authors:** Cédric Lukas, Julia Mary, Michel Debandt, Claire Daïen, Jacques Morel, Alain Cantagrel, Bruno Fautrel, Bernard Combe

**Affiliations:** 1 Rheumatology Department, CHU Montpellier, Montpellier University, Montpellier, France; 2 Rheumatology Department, CHU Martinique, Pierre Zobda-Quitman Hospital, 97200 Fort-de-France, Martinique, French West Indies France; 3 Rheumatology Department, University Paul Sabatier Toulouse III, Purpan Hospital, 31059 Toulouse, France; 4 Sorbonne Université, Institut Pierre Louis d’Epidémiologie et Santé Publique, INSERM UMR S 1136, AP-HP, Groupe hospitalier Pitié Salpêtrière, Service de Rhumatologie, Paris, France

**Keywords:** Rheumatoid arthritis, Seronegative, ESPOIR cohort, DMARD

## Abstract

**Background and objective:**

Early seronegative rheumatoid arthritis (RA) is considered a specific entity, especially regarding diagnostic issues and prognosis. Little is known about its potentially different initial clinical presentation and outcome. We aimed to determine predictors of good response to conventional synthetic disease-modifying anti-rheumatic drugs (csDMARDs) in seronegative RA patients with early inflammatory arthritis.

**Patients and methods:**

Patients from the ESPOIR cohort with early inflammatory arthritis fulfilling the 2010 ACR/EULAR classification criteria for RA despite negativity for both rheumatoid factor and anti-CCP antibodies. The primary endpoint was a good or moderate EULAR response assessed after 1 year of follow-up, given at least 3 months of treatment with a csDMARD. Secondary objectives were to compare the early therapeutic response to methotrexate (MTX) and leflunomide (LEF) versus other csDMARDs (hydroxychloroquine, sulfasalazine) and to identify factors associated with functional disability (Health Assessment Questionnaire-Disability Index [HAQ-DI] > 0.5 at 1 year) and structural progression (van der Heijde-modified total Sharp score > 1 and > 5 points at 1 year). Logistic regression analysis was used to determine independent predictors of outcomes.

**Results:**

One hundred seventy-two patients were analyzed. Overall, 98/172 (57%) patients received MTX during the first year of follow-up. A good or moderate EULAR response at 1 year was associated with early use of csDMARDs (i.e., within 3 months after the first joint swelling) on univariate and multivariable analysis (odds ratio = 2.41 [95% confidence interval 1.07–5.42], *p* = 0.03). Response rates were not affected by other classical prognostic factors (i.e., baseline DAS28). Presence of erosions at baseline was associated with Sharp score progression > 1 point and > 5 points (both *p* = 0.03) at 1 year. HAQ-DI ≥ 1 at inclusion and active smoking were significantly associated with HAQ-DI > 0.5 at 1 year.

**Conclusion:**

Our results suggest that delay in initiation of csDMARD more than baseline clinical, biological, or imaging features predominantly affects the outcome in early seronegative RA. These findings confirm that the usual therapeutic concepts in RA (early treatment, tight control, and treat-to-target) should be applied similarly to both seropositive and seronegative disease forms.

**Trial registration:**

ClinicalTrials.gov: NCT03666091. Registered September 11, 2018.

## Background

Rheumatoid arthritis (RA) is a chronic autoimmune disease affecting about 0.4% of the general population [1]. Rheumatoid factor (RF) and anticitrullinated protein antibodies (ACPA) are the most relevant antibodies associated with RA, and their testing is valuable early in the disease course. However in early disease, antibody detection has been reported as low as 50% [2].

RF and ACPA status are important for both the diagnosis and prognosis of RA [3, 4]. Seropositive RA, particularly ACPA-positive status, is associated with increased likelihood of the development of erosions and further radiographic progression [5–7]. Less is known about the clinical presentation and outcomes of seronegative RA, and studies are disparate given that seronegative RA is more challenging to classify and may indeed represent a heterogeneous population.

Early and intensive treatment of RA with disease-modifying antirheumatic drugs (DMARDs) is clinically beneficial [8, 9]. The difficulty in reliably diagnosing RA presumably explains the discrepancy between general guidelines and daily practice: although methotrexate (MTX) should be prescribed as soon as RA is diagnosed, it is less often prescribed to “seronegative” patients and mostly as a single treatment [10]. Furthermore, the association between the presence of autoantibodies and response to treatment is controversial [11–13]. Because seronegative RA is thought to represent a separate entity with presumably different pathogenesis and less severe disease, assessing the differences in response to treatment with seronegative RA would be useful for physicians.

To our knowledge, no study has specifically assessed seronegative patients in terms of response to treatment. Therefore, we aimed to describe characteristics of seronegative RA disease at presentation and assess factors associated with good response to specific treatment and whether the choice of the first prescribed DMARD might influence the short-term clinical evolution (at 1 year).

## Methods

### Objectives

For this study, we used data from a French longitudinal prospective cohort of adult patients with early arthritis, the Etude et Suivi des POlyarthrites Indifferenciées Récentes (ESPOIR) cohort [14], to determine predictors of good response to conventional DMARDs at 1 year in seronegative RA patients. Secondary objectives were to compare the early therapeutic response to MTX and leflunomide (LEF) versus other conventional synthetic DMARDs (csDMARDs; hydroxychloroquine, sulfasalazine) and to identify factors associated with functional disability (Health Assessment Questionnaire-Disability Index [HAQ-DI] > 0.5 at 1 year) and structural progression (van der Heijde-modified total Sharp score [mTSS] > 1 point and > 5 points at 1 year).

#### Study population

The ESPOIR cohort is a nationwide prospective cohort study of adults conducted under the umbrella of the French Society of Rheumatology. The cohort was constituted by asking general practitioners and rheumatologists to refer patients with early arthritis to one of the 14 university hospitals participating in the ESPOIR cohort project. The protocol has been described in detail elsewhere [14] (ClinicalTrials.gov NCT03666091). Briefly, patients were eligible if they had a definite or probable clinical diagnosis of RA or a diagnosis of undifferentiated arthritis with potential for progression to RA. Patients were included if they were 18 to 70 years old and had swelling of ≥ 2 joints for 6 weeks, symptom duration < 6 months, and no prior treatment with DMARDs or glucocorticoids. Patients with another definite diagnosis of an inflammatory rheumatic disease at the baseline visit were excluded. Included patients were evaluated every 6 months for 2 years, then once a year for at least 10 years. Each center acted as an observational center and did not interfere with patient treatment unless it was in charge of the patient. The patients were routinely monitored and followed by private rheumatologists in the geographic area. Between November 2002 and April 2005, 813 consecutive patients were included in the ESPOIR cohort. Patients were included in the current analysis if they fulfilled the 2010 American College of Rheumatology/European League Against Rheumatism (ACR/EULAR) classification criteria for RA [15], were seronegative for both ACPA and RF, and had been prescribed at least 1 DMARD over the first year period of follow-up; all other individuals, defined as seropositive, were excluded.

#### Baseline assessment

We collected data on demographics (age, sex); socioeconomic status; education (primary, middle, or high school or university); tobacco exposure; duration of symptoms at first visit (defined by the first fixed swollen joint); clinical features [number of swollen joints (0–28); number of tender joints (0–28); visual analog scale (VAS; 0–100) overall assessment by the physician; Disease Activity Score in 28 joints (DAS28) [16]; functional disability by the HAQ-DI [17]]; therapeutic regimen; biological features [including erythrocyte sedimentation rate (ESR; mm/h); level of C-reactive protein (CRP; mg/l) by standard laboratory methods]; radiographs of hands, wrists, and feet in the posteroanterior view; and therapeutic regimen. Radiographs of the hands, wrists, and feet were scored for the presence of erosions and joint space narrowing according to the mTSS [18] by an experienced rheumatologist who was blinded to the patient’s other data.

#### Follow-up assessment and outcomes

The primary endpoint was a EULAR response classified as good or moderate versus none evaluated at 1 year, given at least 3-month csDMARD treatment over the first year of follow-up had been followed. Patients with at least 3 months of a first csDMARD but who switched to a second drug were considered non-responders to the first treatment. Because the reason for the switch was not specifically collected in ESPOIR, we considered that a very early withdrawal (within 1 month) of a csDMARD was presumably due to intolerance or side effects. In the latter situation, the second csDMARD used was considered the first-line therapy. We also considered patients with more than 1 month but no longer than 3-month treatment with a csDMARD as non-responders. All patients were followed for at least 1 year. Radiographs were obtained and scored at 12 months in a chronological order by using the same technique. Radiographic progression was defined as an increase of at least 1 point of the mTSS (the smallest detectable change derived from this scoring) or the erosion score assessed at baseline and after 12 months [19].

#### Statistical analysis

Baseline characteristics and disease evolution are described with mean ± SD or frequency (%) as appropriate. Baseline characteristics and disease course were compared between good or moderate EULAR responders and non-responders by the Mann-Whitney *U* test (for numerical data) and Fisher exact test (for categorical data). Logistic regression analyses were used to determine relevant independent baseline variables, estimating odds ratios (ORs) and 95% confidence intervals (CIs). The explanatory variables included in the logistic regression model were derived from results of univariate analyses. Significance was defined as *p* < 0.05 for variables in the final multivariable model. A similar approach was used to analyze the association of baseline variables with the secondary outcomes (i.e., radiographic progression [change in mTSS at least 1 point and at least 5 points] and functional impairment at 1 year [HAQ-DI > 0.5]). To compare the early therapeutic response to MTX and LEF versus other csDMARDs (hydroxychloroquine, sulfasalazine), a propensity score was used to reduce confounding by indication bias. Indeed, the likelihood of prescribing MTX or LEF rather than hydroxychloroquine or sulfasalazine is influenced by the appreciation by the rheumatologist of the disease severity and activity at baseline. The propensity score was computed by using a multivariable logistic regression model. All demographic and disease characteristics at baseline were used as covariates in the model: sex, age at inclusion, ESR, CRP level, tender and swollen joint count, disease activity, VAS score, HAQ-DI, erosive disease, erosion score, and joint narrowing score. As sensitivity analyses, we conducted the same analysis plan in the entire ESPOIR cohort, restricted to patients fulfilling ACR/EULAR 2010 criteria for RA and having been treated by at least 1 DMARD in their first year of follow-up. We also tested alternative outcome measures than EULAR response in seronegative patients, with DAS28 remission and DAS28 remission/low disease activity at 12 months being used. Statistical analysis involved using SPSS v15. *P* < 0.05 was considered statistically significant.

## Results

### Patient characteristics

**Fig. 1:**
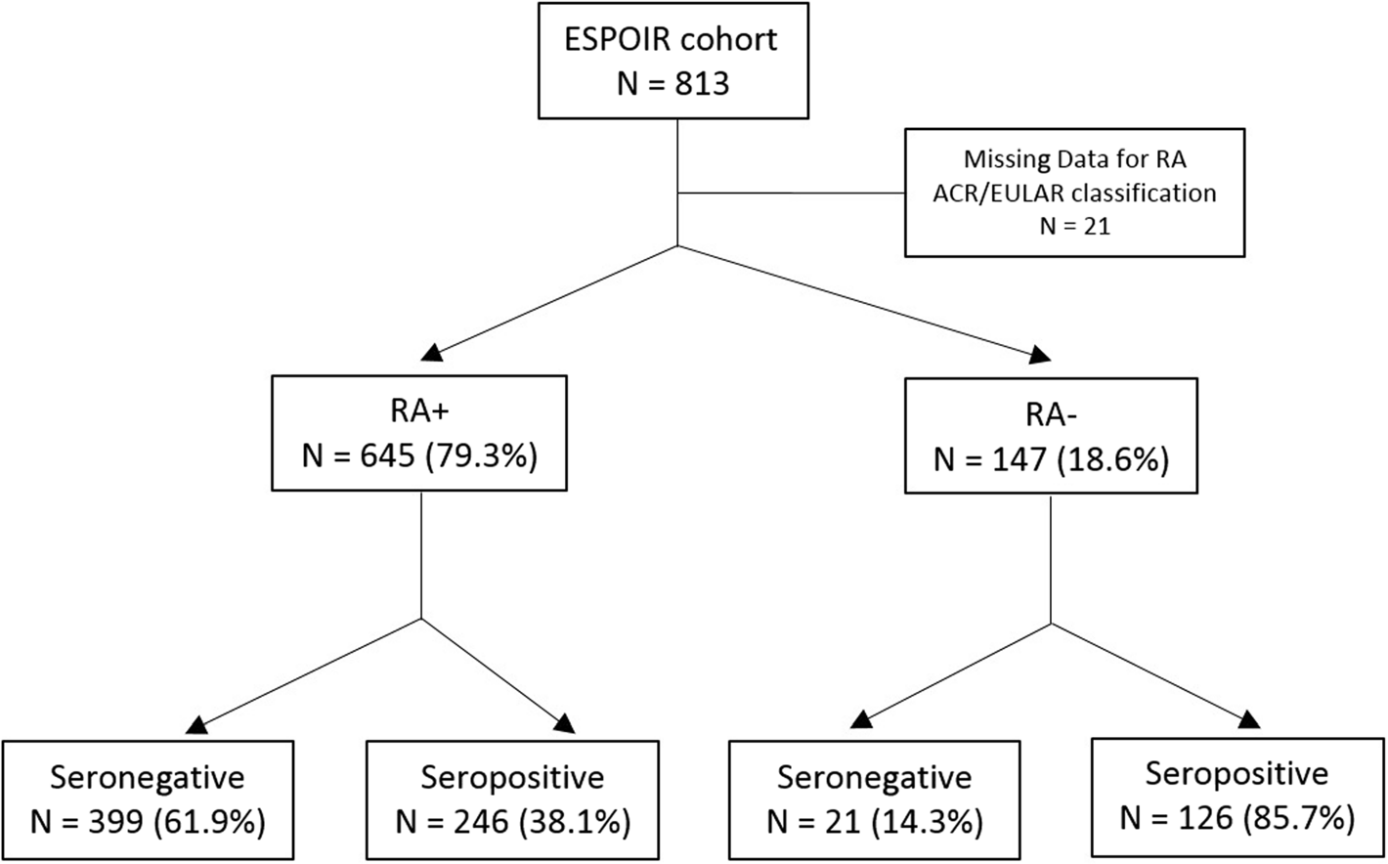
Flowchart of ESPOIR cohort’s patients. RA, rheumatoid arthritis; RA+, ACR/EULAR classification for RA fulfilled; RA−, ACR/EULAR classification for RA not fulfilled; seronegative, both rheumatoid factor- and CCP-tests negative; seropositive, at least 1 positive test from rheumatoid factor- and CCP-tests

From the 813 patients included in the ESPOIR cohort, 24 had missing data regarding RF-and CCP-tests. Of the 789 with available data, 384 (48.7%) were “seronegative” for both autoantibodies (RF and ACPA), while 405 had at least 1 positive test from RF and CCP tests. Twenty-one patients could not be classified according to ACR EULAR 2010 criteria due to missing data (i.e., 3 had sufficient criteria even without information regarding serologic status to be classified having RA). From these 792 patients, 645 (79.3%) were classified RA, 399 (61.9%) of them being seropositive, and 246 (38.1%) seronegative. From the 147 not being classified RA, 21 (14.3%) were seropositive and 126 (85.7%) seronegative (Fig. 1). We included 172 patients in the current study (*n* = 58 had not received a csDMARD during the first year, *n* = 19 were lost to follow-up, *n* = 29 had missing data). Most characteristics were similar between excluded and included patients, except for baseline HAQ-DI, which was slightly lower for excluded than included patients (mean ± SD 0.89 ± 0.67 vs 1.1 ± 0.68). Baseline characteristics are shown in Table 1. The mean ± SD age was 49.5 ± 12.8 years, and 80.8% of patients were females. At inclusion, the mean ± SD number of swollen joints was 9.0 ± 5.5 and number of tender joints 11.7 ± 7.1. Mean DAS28 score at baseline was 5.5 ± 1.1. MTX was the most commonly prescribed first DMARD (98/172 [57%] patients).

**Table 1:** Baseline characteristics of the study population (*n* = 172)

Characteristic	
Women, no./total (%)	139/172 (80.8)
Age, mean ± SD, years	49.5 ± 12.8
Smoking status, no. (%)	
Current smoking	46 (26.7)
Past smoking	42 (24.4)
Never smoked	93 (54.1)
DAS28, no. (%)	
Low disease activity	1 (0.6)
Moderate disease activity	70 (40.7)
High disease activity	101 (58.7)
Tender joint count, mean ± SD	9 ± 5.5
Swollen joint count, mean ± SD	11.7 ± 7.1
HAQ-DI, mean ± SD	1.1 ± 0.7
ESR (mm/h), mean ± SD	25.2 ± 23.6
CRP (mg/dl), mean ± SD	23.2 ± 42.8
mTSS, mean ± SD	5.5 ± 7.0
Treatment, no. (%)	
Methotrexate	98 (57)
Leflunomide	12 (7)
Sulfasalazine	22 (12.8)
Hydroxychloroquine	37 (21.5)
Cyclophosphamide	1 (0.6)
Biologic DMARD	2 (1.2)

*CRP* C-reactive protein, *DAS28* Disease Activity Score in 28 joints, *DMARD* disease-modifying antirheumatic drug, *ESR* erythrocyte sedimentation rate, *HAQ* Health Assessment Questionnaire-Disability Index, *mTSS* van der Heijde-modified total Sharp score

### Predictors of good or moderate EULAR response at 12 months

At 1 year, 114/172 (66%) patients showed a good or moderate EULAR response. On univariate analysis, a good or moderate EULAR response was significantly associated with swollen joint count (≥ 7), early treatment (started within 3 months after the date of first reported synovitis), ESR, CRP level, and HAQ-DI ≥ 1 (*p* < 0.05 for all comparisons).

On multivariable analysis (Table 2), a good or moderate EULAR response was associated with only early use (within 3 months) of csDMARDs (OR 2.41 [95% CI 1.07–5.42], *p* = 0.03).

**Table 2:** Multivariable analysis of factors associated with good or moderate EULAR response at 1 year (*n* = 172)

	OR	95% CI	*p*
Age > 49 years	0.82	0.35–1.89	0.64
Sex (female)	1.52	0.51–4.53	0.45
Education level			
Primary school	0.29	0.08–1.03	0.06
Middle school	0.53	0.19–1.46	0.22
High school	ref	–	0.23
University	0.73	0.25–2.15	0.56
Smoking status			
Current smoking	ref	–	0.70
Past smoking	0.82	0.24–2.77	0.75
Never smoked	1.23	0.42–3.65	0.71
DAS28 level			0.71
Tender joint count ≥ 10	1.21	0.47–3.14	0.68
Swollen joint count ≥ 7	0.94	0.33–2.69	0.91
Rheumatologist global assessment ≥ 66/100 (VAS)	1.15	0.51–2.58	0.74
Early DMARD treatment^1^	2.41	1.07–5.42	0.03
HAQ-DI ≥ 1	1.41	0.67–2.98	0.37
ESR ≥ 15 mm/h	1.42	0.66–3.03	0.37
CRP level ≥ 7 mg/l	0.71	0.32–1.61	0.42
mTSS (per unit)	1.00	0.94–1.07	0.96
mTSS	0.87	0.30–2.52	0.79

*CRP* C-reactive protein, *DAS28* Disease Activity Score in 28 joints, *ESR* erythrocyte sedimentation rate, *HAQ-DI* Health Assessment Questionnaire-Disability Index, *mTSS* van der Heijde-modified total Sharp score, *VAS* visual analog scale, *OR* odds ratio, *95% CI* 95% confidence interval

^1^Within 3 months after the first joint swelling

### Association between early therapeutic response to MTX and LEF versus other csDMARDs (hydroxychloroquine, sulfasalazine)

After adjustment for propensity of patients to receive MTX, LEF, or the 2 drugs combined versus other csDMARDs, we found no significant difference in response rates at 1-year follow-up visit (Additional file 1: Table S1).

### Predictors of radiographic outcome at 12 months

Radiographic data were available for 149 patients at 1 year. In total, 15 (10%) and 9 (6%) showed a progression of at least 1 point and 5 points, respectively, in mTSS at 1 year. On multivariable analysis, the probability of radiographic progression by a least 1 mTSS point at 1 year was significantly increased in patients with erosions on baseline radiographic assessment and was decreased in those with > 10 tender joints. The only characteristic associated with a radiographic progression of at least 5 mTSS points at 1 year was the presence of erosions at baseline (OR = 5.42 [95% CI 1.14–25.7], *p* = 0.03).

### Factors associated with functional disability

HAQ-DI data were available for 150 patients at 1 year. HAQ-DI was > 0.5 for 72 (48%) patients at 1 year. On multivariable analysis, functional disability (defined by a HAQ-DI > 0.5) at 1 year was significantly associated with increased baseline functional disability defined as HAQ-DI > 1 (OR = 6.59 [95% CI 3.29–13.2], *p* < 0.001), female sex (0.28 [0.10–0.79], *p* = 0.02), ESR > 15 (0.45 [0.20–0.98], *p* = 0.05), and active smoking status (2.59 [1.00–6.69], *p* = 0.05) [20].

### Results in entire ESPOIR cohort and alternative outcome measures

The sensitivity analysis in the entire ESPOIR cohort was applied to the 522 patients fulfilling the aforementioned selection criteria, with 69 having missing data for at least 1 critical variable in our analysis plan and 453 thus finally included. The EULAR good/moderate response was obtained by 369 (81.5%) of these included patients. The main result was confirmed in this larger sample of patients, with a significant association between the early DMARD start and a favorable therapeutic response: OR 2.29 [1.27–4.12], *p* = 0.006. The larger sample size revealed another significantly associated variable, with patients having a higher CRP (≥ 7 mg/L) also showing a higher likelihood of favorable outcome: OR 1.83 [1.13–2.97], *p* = 0.015. Interestingly, the addition of the variable “seronegative yes/no” in the list of candidate variables when building the multivariate logistic model showed no relevant association with the therapeutic outcome (variable not retained by the stepwise model). When other outcome measures of the therapeutic response were applied to the seronegative patients (DAS28 remission or DAS28 remission/low disease activity), the association with the early DMARD start remained in the final multivariate model, although at a statistically non-significant level (data not shown).

## Discussion

With an inception cohort of patients with early inflammatory arthritis (the ESPOIR cohort), we described the characteristics and evolution over the first year of follow-up of those fulfilling the ACR/EULAR criteria for RA but with no detectable RF or anti-CCP antibodies (“seronegative RA”). Overall, 246 of the 803 included patients fulfilled criteria for RA despite a seronegative status. The most important predictor of good therapeutic response in these patients was the early introduction (within 3 months after symptom onset) of a csDMARD (MTX being the most commonly used). Response rates did not differ across available csDMARDs after adjustment for potential baseline prognostic factors and thus expected confounding by indication bias.

According to common therapeutic recommendations [21], MTX was the first-line therapy for most patients in our cohort (57%), despite being negative for serologic factors of RA, which are considered relevant elements for diagnostic certainty and potentially severe prognosis. With regard to other similar studies, MTX was often used as first-line treatment [22, 23], which is probably due to the time frames, the patients in our study cohort having been enrolled more recently (years 2002–2005).

Overall, 66% of our patients had a good or moderate EULAR response to the first DMARD used at 1-year follow-up. Although we cannot directly compare to previous studies because of different outcomes used and potentially different inclusion criteria or recruitment methods, previous studies also showed a favorable therapeutic response to traditionally used medications in seronegative RA patients [22, 24, 25].

With regard to factors associated with a more favorable outcome and independent of other measurable and collectable characteristics, the only relevant prognostic marker of a good therapeutic response was the early introduction (within 3 months after symptom onset) of the DMARD. This finding was already reported for the entire ESPOIR cohort [19], which confirms that the serologic status of a patient should not imply a major change in therapeutic management, provided the diagnostic approach has been appropriately conducted. Other studies have shown similar results and confirmed the benefit of early treatment start, but our study is the first time that this finding was replicated in seronegative patients [26–29].

Because seronegativity is supposed to be associated with better overall prognosis of RA, we expected that these patients would receive less intensive treatment than patients with detectable levels of RF and/or anti-CCP antibodies [30–32]. However, a similar proportion of patients had received MTX or LEF as first-line therapy in the entire ESPOIR cohort as in this sub-population with no detectable serological biomarker of the disease [19]. Other csDMARDs were also alternatively prescribed in these conditions, and we investigated whether this prescription might have measurable consequences in short-term prognosis (first-year therapeutic response in particular). After adjustment for known and collected factors associated with potentially worse prognosis and thus a higher propensity for the rheumatologist to prescribe more intensive treatments, the response rates did not differ in our patients with seronegative RA who received anchor drugs or “second-choice drugs” such as sulfasalazine or hydroxychloroquine. However, the method applied (propensity-score adjustment) as well as the limited number of patients in the second group requires cautious interpretation, and we report our observations without drawing any definite conclusions regarding the optimal choice of the first csDMARD in this context. In another study, a similar response rate was also reported in anti-CCP–negative RA patients receiving MTX or a combination of csDMARDs [7].

## Conclusions

In terms of prognostic factors associated with RA structural progression, we confirmed that the strongest predictor of further joint degradation remains the presence of early erosions seen on hands and feet radiographs [33]. These results confirm that despite the overall better expected prognosis with seronegative versus seropositive RA, in particular anti-CCP positivity, careful and complete examination of every patient is crucial, before the appropriate and optimal therapeutic management can be determined.

Several limitations of our study must be acknowledged. First, because our main objective was to determine factors associated with a favorable therapeutic response in seronegative RA, we restricted our analyses to patients who received a csDMARD over the follow-up period. In the ESPOIR cohort, 246 patients had seronegative RA according to ACR/EULAR criteria, but 58 did not receive any DMARD during the first year of follow-up and were thus not included. Presumably for these patients, their rheumatologist considered that they had sufficiently mild disease or potentially self-remitting disease to avoid a specific drug prescription on the basis of clinical, biological, or radiological data and short-term disease course. Therefore, our results should be applied to patients fulfilling RA criteria and justifying DMARD initiation based on the rheumatologist’s opinion. Indeed, the observed disease activity was higher on average in our population than in other reported cohorts of seronegative RA patients [7, 22, 24, 25]. It is important to note that for fulfilling ACR/EULAR 2010 RA criteria, patients with seronegative RA should have a higher number of joints involved than seropositive patients, though high disease activity is an important prognostic factor in early arthritis, and thus a potential confounding factor in our work.

In conclusion, we confirmed that seronegative RA does not greatly differ from overall RA in terms of both therapeutic response and structural and functional prognosis and, most importantly, that the usual and consensually recommended concepts of therapeutic management should not be applied differently in these patients. Indeed, despite a generally more favorable expected prognosis with seronegative RA, the positive impact of an appropriate initial management remains measurable, with the concept of the “window of opportunity” being confirmed in this sub-population, pleading for an early start of a DMARD, ideally within 3 months after symptom onset.

## Supplementary information

**Additional file 1:** **Table S1.** Impact of the type of prescribed disease-modifying anti-rheumatic drug (DMARD) on 1-year EULAR response rate: multivariable analyses adjusted for the propensity of receiving methotrexate or leflunomide as first-line therapy.

## Data Availability

The data that support the findings of this study are available from the ESPOIR scientific committee but restrictions apply to the availability of these data, which were used under license for the current study, and so are not publicly available. Data are however available from the authors upon reasonable request and with permission of the ESPOIR scientific committee.

## Abbreviations

ACPA: Anti-citrullinated peptide antibodies
ACR/EULAR: American College of Rheumatology/European League Against Rheumatism
CRP: C-reactive protein
DAS28: Disease Activity Score in 28 joints
csDMARDs: Conventional synthetic disease-modifying antirheumatic drugs
ESR: Erythrocyte sedimentation rate
HAQ-DI: Health Assessment Questionnaire-Disability Index
LEF: Leflunomide
mTSS: Van der Heijde-modified total Sharp score
MTX: Methotrexate
NSJ: Number of swollen joints
NTJ: Number of tender joints
RA: Rheumatoid arthritis
RF: Rheumatoid factor
VAS: Visual analog scale
