# Supplementary material for: Predictors of good response to conventional synthetic DMARDs in early seronegative rheumatoid arthritis: data from the ESPOIR cohort
Source: Arthritis Res Ther. 2019 Nov 15;21:243. doi: 10.1186/s13075-019-2020-x (PMC6858774; doi:10.1186/s13075-019-2020-x)
Supplement: Supplementary file 1 — Additional file 1: Table S1. Impact of the type of prescribed disease-modifying anti-rheumatic drug (DMARD) on 1-year EULAR response rate: multivariable analyses adjusted for the propensity of receiving methotrexate or leflunomide as first-line therapy. [file 13075_2019_2020_MOESM1_ESM.docx]

**Table S1. Impact of the type of prescribed disease-modifying anti-rheumatic drug (DMARD) on 1-year EULAR response rate: multivariable analyses adjusted for the propensity of receiving methotrexate or leflunomide as first-line therapy.**

| Type of first DMARD prescribed | OR [95% CI] | p |
| --- | --- | --- |
| Methotrexate (versus HCQ, SSZ, no DMARD) | 1.62 [0.78-3.39] | 0.20 |
| Methotrexate or leflunomide (versus HCQ, SSZ, no DMARD) | 1.27 [0.59-2.75] | 0.55 |

HCQ=hydroxychloroquine, SSZ=sulfasalazine, DMARD=disease modifying conventional drug, OR=odds ratio, 95% CI=95% confidence interval
